# The Volatile Compounds Change during Fermentation of *Saccharina japonica* Seedling

**DOI:** 10.3390/foods13131992

**Published:** 2024-06-24

**Authors:** Jingni Gong, Xiaolin Wang, Hui Ni, Yonghua Wang

**Affiliations:** 1 School of Food Science and Engineering, South China University of Technology, Guangzhou 510640, China; 2 College of Food and Biological Engineering, Jimei University, Xiamen 361021, China; 3 Fujian Provincial Key Lab of Food Microbiology and Enzyme, Jimei University, Xiamen 361021, China; 4 School of Marine Biology, Xiamen Ocean Vocational College, Xiamen 361000, China; 5 Guangdong Youmei Institute of Intelligent Bio-Manufacturing, Foshan 528225, China

**Keywords:** *Saccharina japonica* seedling, aroma, volatile compounds, *Saccharomyces cerevisiae*, GC-MS

## Abstract

It is important to eliminate the fishy odor and improve the aroma quality of seafood. In this study, the *Saccharina japonica* (*S. japonica*) seedling, which is a new food material, was investigated for the effects of fermentation with *Saccharomyces cerevisiae* (*S. cerevisiae)* through sensory evaluation, GC–MS, and odor activity value (OAV) analysis. GC–MS analysis revealed the presence of 43 volatile compounds in the unfermented *S. japonica* seedling, with 1-octen-3-ol, hexanal, and trans-2,4-decadienal identified as the main contributors to its fishy odor. After fermentation with *S. cerevisiae*, 26 volatile compounds were identified in the *S. japonica* seedling. Notably, the major malodorous fish compounds, including 1-octen-3-ol, hexanal and trans-2,4-decadienal, were no longer present. The results indicate that fermentation with *S. cerevisiae* is an effective method for removing fishy malodor compounds and enhancing the volatile components with fruity, sweet, green, and floral notes in the *Saccharina japonica* seedling. This process facilitates the elimination of fishy malodor and enhance the fruity, sweet, green, and floral notes of *S. japonica* seeding and other seaweeds.

## 1. Introduction

*Saccharina japonica* (J.E. Areschoug) C.E. Lane, C. Mayes, Druehl & G.W. Saunders, also known as kelp and kombu, is a type of brown algae. *S. japonica* is a marine product that is rich in nutrients and contains a variety of functional substances, including protein, amino acids, polysaccharides, minerals, and vitamins [1,2]. *S. japonica* is particularly rich in iodine, and consuming this marine product could help prevent thyroid diseases [3,4]. In addition, *S. japonica* exhibits a variety of functional properties, including antioxidant, anti-inflammatory, hypoglycemic, and antihypertensive effects [5,6,7,8,9]. Therefore, *S. japonica* represents an important marine alga with the potential to provide health benefits to humans.

The aroma quality of food has a noticeable effect on the consumption preference of consumers. However, *S. japonica* products always have a strong fishy odor, which becomes the main barrier to human consumption [10,11]. In order to increase the consumption of *S. japonica*, it is essential to eliminate its fishy odor [12]. The methods of deodorization mainly include physical, chemical, and biological methods [13]. Compared with physical and chemical methods, biological methods are attracting more and more attention from researchers and manufacturers, due to their safety, environmental friendliness, and ease of implementation. Seo et al. [14] proposed fermenting *S. japonica* extract with *Aspergillus oryzae* to reduce its off-flavor. Although researchers have developed effective deodorization processes to remove the off-flavor from extracts and blends of *S. japonica*, few studies have been conducted to eliminate the fishy malodor from the foods made from *S. japonica* [14].

Recently, fermentation has been proposed to eliminate the off-flavors of *S. japonica* and improve the quality of *S. japonica* food products [15]. Nie et al. [16] demonstrated that the fermentation of *S. japonica* resulted in the reduction of fishy odorants, including hexanal and nonanal. Zhu et al. [17] demonstrated that fermentation with *Saccharomyces cerevisiae* could reduce 91% of unsaturated aldehydes from *S. japonica*. In addition, *Lactobacillus plantarum* and *Acetobacter pasteurianus* have also been used to ferment foods with desirable aromas [18,19]. In comparison, fermentation with *Saccharomyces cerevisiae* not only reduces fishy odors but also enhances fruit and floral aromas [17]. Therefore, researchers prefer to use *S. cerevisiae* to enhance the flavor of plant, seaweed, and fish products [20,21], which could be a practical method to enhance the flavor quality of food products made from *S. japonica*.

The *S. japonica* seedling is the *S. japonica* harvested at the early growth stage. It has higher nutritional and bioactivity values [22], making it increasingly important in food processing in recent years. However, *S. japonica* seedlings have a fishy odor, which limits consumer acceptance. Therefore, this study aimed to investigate the effectiveness of fermentation with *Saccharomyces cerevisiae* in removing the fishy odor from *S. japonica* seedlings. This study contributes to the understanding of the fishy odor of the novel food material *S. japonica* seedlings and provides a reference for enhancing the fruity, sweet, green, and floral notes of food products derived from *S. japonica* seedlings.

## 2. Materials and Methods

### 2.1. Chemicals

Standard 1-octen-3-ol (98%), 2-octen-1-ol (97%), 1-octanol (≥99%), anethol (≥99%), phenethyl alcohol (≥99%), 1-nonanol (≥98%), decyl aldehyde (analytical standard), undecanal (analytical standard), 1-octen-3-one (≥98%), 3-acetyl-2-octanone (≥98%), β-ionone (≥96%), 1,3-octadiene (≥95%), undecene (97%), 4-methyl-1-hendecene (97%), 8-heptadecene (98%), hexyl acetate (analytical standard), phenethyl acetate (analytical standard), hexyl butyrate (98%), ethyl caprate (≥99%), ethyl laurate (analytical standard), methyl palmitate (analytical standard), (E)-2-nonenal (≥95%), β-cyclocitral (≥95%), hexanal (95%), nonanal (96%), limonene (97%), and α-terpinene (≥95%) were purchased from Sigma Aldrich (St. Louis, MO, USA). Standard 2,4-dimethylbenzaldehyde (97%) and 2-pentylfuran (≥98%) were obtained from Alfa Aesar Co., Ltd. (Heysham, UK). A standard series of C_8_–C_20_ alkanes (analytical standard) was used for retention index (RI) determination, and the internal standard cyclohexanone was purchased from Sigma Co., Ltd. (St. Louis, MO, USA).

### 2.2. Preparing the Algae and Yeast

A *Saccharina japonica* seedling was obtained from the FuJian province of China in 2020. It was subsequently divided into 2 cm × 2 cm pieces. *Saccharomyces cerevisiae* CICC 1464 was purchased from the China Food and Fermentation Industry Research Institute Co., Ltd. (Beijing, China). The *S. cerevisiae* strain was activated in a 250 mL flask containing 50 mL of malt extract medium (malt extract 130 g/L and chloramphenicol 0.1 g/L) using a ZQZY-CF shaker (Shanghai Chuzhi Biotechnology Co., Ltd. (Shanghai, China) at 180 rpm and 28 °C for 3 days. The *S. cerevisiae* cells were harvested by centrifugation at 1500 rpm and 4 °C for 5 min.

### 2.3. Fermentation Procedure

The fermentation was conducted following the methods described in the literature, with minor modifications [23]. In detail, the yeast seed was adjusted to an optical density at 600 nm (OD_600_) of 0.8 with sterilized water. Ten grams of the *S. japonica* seedling was mixed with 100 mL of activated *S. cerevisiae* solution, followed by fermentation at 28 °C and 150 rpm for 120 min. The fermented *S. japonica* seedling was then removed and washed three times with 20 times the volume of distilled water. The seaweed was then dried at 40 °C to a moisture content of 9.5%.

### 2.4. Sensory Evaluation of Odor

According to the relevant literature [23] and ISO 13299:2016-05 [24], quantitative descriptive analysis (QDA) was used for the sensory evaluation of the odor. Ten panelists (5 males and 5 females) aged 22–26 years were trained to become familiar with the intensity of “fishy”, “floral”, “green”, “fatty”, “earthy”, “fruity” and “sweet” notes. For the sensory evaluation of the samples, five grams of *S. japonica* seedling was placed in a 100 mL Erlenmeyer flask, and the sensory evaluation was carried out at 26 ± 2 °C in a clean environment. After each sniff, the evaluators needed a 20 s gap with fresh air to refresh their olfactory fatigue. The panelists rated the sample on a scale of 0–9, with 0 representing no perceived attribute intensity and 9 representing very strong attribute intensity. All analyses were conducted in triplicate, and the average value was considered the final score of the sample, which was then visualized on the radar plot. The rights and privacy of all participants were protected during the research. This included ensuring no coercion to participate, providing full disclosure of study requirements and risks, obtaining verbal consent from the participants, refraining from releasing participant data without their knowledge, and allowing the participants to withdraw from the study at any time.

### 2.5. GC-MS Analysis

Thirty milliliters of distilled water and 1 g of *S. japonica* seedling were added to an extraction vial containing 10 µL of the internal standard (cyclohexanone). The sample was equilibrated at 60 °C for 30 min, followed by extraction for 30 min with a 50/30 µm DVB/CAR/PDMS extraction fiber.

A QP2010 gas chromatography–mass spectrometry (GC-MS) system (Shimadzu, Kyoto, Japan) was equipped with an Rtx-5MS column (60 m × 0.32 mm × 0.25 µm, Restek Corporation, Bellefonte, PA, USA). Helium (99.999% purity) was used as the carrier gas. The column flow rate was 3.16 mL/min in splitless injection mode. The injection port temperature was 230 °C. The column temperature was 40 °C at the start, increased to 100 °C at a rate of 6 °C/min, and then further increased to 230 °C at a rate of 5 °C/min and held for 5 min. The temperatures of the ion source and the interface were 220 °C and 250 °C, respectively [23]. The MS was set to electron impact (EI) ionization mode with a mass scan range from 35 to 500 amu, and the electron energy was set at 70 eV. The solvent delay time was 3 min.

Mass spectra were compared to the NIST library (NIST11, NIST11s, FFNSC1.3) to identify relevant molecules. The compounds with a mass spectrometry match greater than 80% were screened out. The identification was then confirmed by combining the base peak, characteristic ion peak, and retention index (RI). The retention index was calculated based on a standard mixture of n-alkanes (C_8_-C_20_) using Equation (1):

(1) $${\mathrm{RI}}_{x}=100n+\;100\;\times \;\left(\frac{{\mathrm{RT}}_{x}-{\mathrm{RT}}_{n}}{{\mathrm{RT}}_{n+1}-{\mathrm{RT}}_{n}}\right)$$

In this equation, RI_x_ is the retention index of the component to be measured, n is the number of carbon atoms in the n-alkanes, RT_x_ is the retention time of each odorant (x), and RT_n_ and RT_n+1_ are the retention times of the n-alkanes eluted before and after the odorant (x) under identical chromatographic circumstances conditions.

The volatile compounds with chemical standards were quantitatively analyzed using their respective calibration curves. The volatile compounds without chemical standards were tentatively identified, and their concentrations were approximately estimated using the internal standard according to Equation (2):

(2) $$C_{x}=\frac{P_{x}\times C_{i}}{P_{i}}$$

In this equation, C_x_ represents the concentration of the compound to be tested, P_x_ represents the peak area of the compound being tested, C_i_ represents the concentration of the internal standard, and P_i_ represents the peak area of the internal standard.

### 2.6. Analysis of odor Activity Value (OAV)

OAV was calculated by dividing the concentration of the volatile compounds by their odor threshold in water using Equation (3). The threshold value was obtained from the literature [25]. If OAV ≥ 1, the compound is considered to be an aroma compound that may be a major contributor.

(3) $$\mathrm{OAV}=C_{i}/{OT}_{i}$$

In this equation, C_i_ is the concentration of volatile compounds and OT_i_ is the odor threshold.

### 2.7. Statistical Analysis

SPSS 19.0 (SPSS, Inc., Chicago, IL, USA) was used to calculate mean and errors and to conduct significance analysis (*p* ˂ 0.05). Analysis of variance (ANOVA) was performed to determine differences in the amounts of the aroma compounds of the quantified volatile compounds before and after fermentation.

## 3. Results

### 3.1. Sensory Evaluation of S. japonica seedling before and after Fermentation

**Figure 1:**
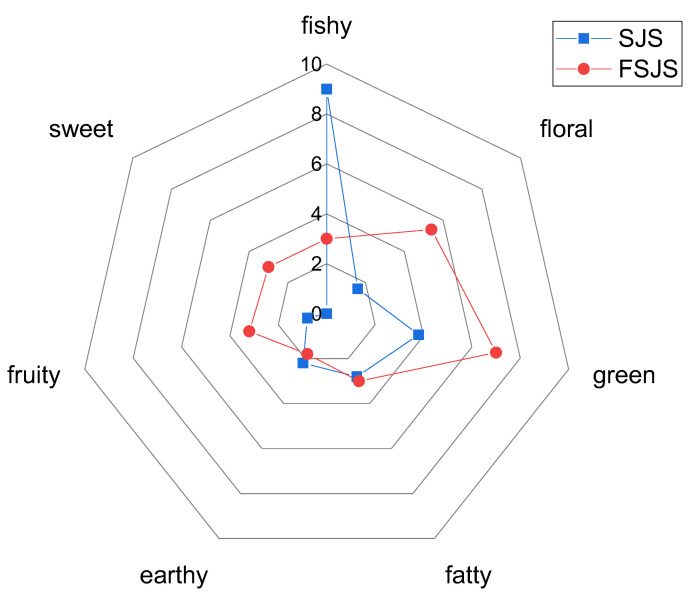
A spider diagram illustrating the sensory characteristics of the *S. japonica* seedling before and after fermentation (SJS refers to the *S. japonica* seedling without fermentation. FSJS is the *S. japonica* seedling after fermentation. 0 refers to unrecognized, 2 refers to can be recognized, 4 refers to weak, 6 refers to middle, 8 refers to strong, 10 refers to very strong).

As shown in Figure 1, the *S. japonica* seedling primarily exhibited a fishy odor, accompanied by subtle notes of green, fatty, earthy, floral, and fruity scents. After fermentation with *S. cerevisiae*, the fishy odor diminished, while the green, floral, sweet, and fruity notes intensified. The results indicated that fermentation with *S. cerevisiae* could enhance the aroma of the *S. japonica* seedling. This finding is consistent with the study of Xu et al. [23].

### 3.2. Qualitative and Quantitative Analysis of Volatile Compounds before and after Fermentation

A total of 43 volatile compounds were detected in the unfermented *S. japonica* seedling, including 6 alcohols, 10 aldehydes, 10 ketones, 4 alkanes, 8 alkenes, 3 esters, and 2 other compounds (Table 1). As shown in Table 1, aldehydes and ketones are the most abundant compounds, followed by alkenes and alcohols. Among the aldehydes, 1-nonanal and hexanal are the most abundant in concentration. Ketones are the second most abundant chemical after aldehydes. Among the ketones, 2,2,6-trimethylcyclohexanone and β-ionone showed relatively high concentrations. Alkenes are the third most abundant chemical after ketones. Among the ketones, 3,5,5-trimethyl-2-hexene and 8-heptadecene exhibited the highest relative abundance in concentration. As for the alcohols, 1-octen-3-ol had the highest concentrations. These findings are consistent with those of Zhu et al. [17].

**Figure 2:**
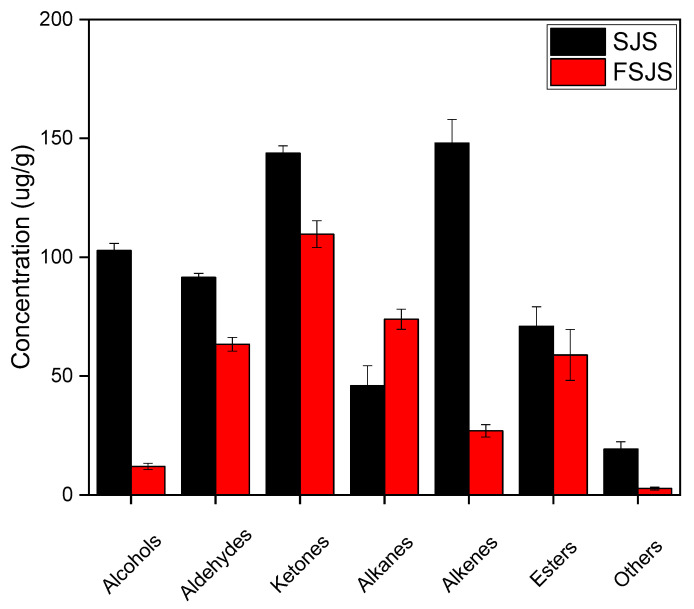
Volatile compound concentrations in *S. japonica* seedling before and after fermentation by *S. cerevisiae*. SJS means unfermented *S. japonica* seedling; FSJS means fermented *S. japonica* seedling.

The fermented *S. japonica* seedling was found to contain 26 volatile compounds (Table 1), including 2 alcohols, 6 aldehydes, 2 ketones, 8 alkanes, 3 alkenes, 4 esters, and 1 other compound. Fermentation affects the concentration of volatile compounds. As shown in Figure 2, the quantities of alcohols, aldehydes, alkanes, esters, ketones, and others decrease. Among these, alcohols and alkenes showed a significant decrease. Alkanes are the only volatile compounds that behaved differently, increasing by almost a third. A comparative analysis of the volatile compounds present in the *S. japonica* seedlings before and after fermentation revealed significant variations in the composition of alcohols, aldehydes, and ketones (Table 1). For example, the content of alcohols such as 1-octen-3-ol, 2-octen-1-ol, 1-octanol, cis-anethol, trans-2-decen-1-ol, and 1-nonanol completely disappeared after fermentation and was replaced by phenethyl alcohol and cedrol. The content of aldehydes, such as hexanal, 2-nonenal, 2,4-dimethylbenzaldehyde, undecanal, and trans-2,4-decadienal, was completely depleted, while the amount of 1-nonanal decreased from 47.4 ± 1.7 to 11.6 ± 1.5 µg/g after fermentation. Most of the ketones disappeared after fermentation, such as 1-octen-3-one, 2,2,6-trimethylcyclohexanone, 3-methyl-2-cyclohexen-1-one, 3,5-octadien-2-one, 3-acetyl-2-octanone, 2,6,6-trimethyl-2-cyclohexanedione, 4-(2,6,6-trimethylcyclohexen-1-yl) butan-2-one, and 5,9-undecadien-2-one, 6,10-dimethyl.

### 3.3. OAV Analysis of Volatile Compounds of S. japonica Seedling before and after Fermentation

The odor activity value (OAV) is the ratio of the compound concentration to its flavor threshold [26]. An OAV ≥ 1 indicates that the compound is a significant contributor to the overall odor [27]. As shown in Table 2, the *S. japonica* seedling before fermentation had 14 compounds with OAV ≥ 1, including 1-octen-3-ol, 2-octen-1-ol, 1-octanol, 1-nonanol, hexanal, 1-nonanal, 2-nonenal, 2,4-dimethylbenzaldehyde, decyl aldehyde, β-cyclocitral, trans-2,4-decadienal, dodecyl aldehyde, β-ionone, and 2-pentylfuran. Among these compounds, 1-octen-3-ol, 1-nonanal, and trans-2,4-decadienal, which had fishy odors, had the highest OAV values. Obviously, these three compounds are the main contributors to the fishy odor of the *S. japonica* seedling. This result is consistent with the findings of Nie et al. [16] and Xu et al. [23].

After fermentation, the *S. japonica* seedling had 7 compounds with OAV ≥ 1, including 1-nonanal, decyl aldehyde, β-cyclocitral, dodecyl aldehyde, and β-ionone. Among these volatiles, 1-nonanal exhibited earthy and fatty odors; decyl aldehyde had ocean, cucumber, and herbal scents; β-cyclocitral emitted liquorice, fruity, and fresh aromas; dodecyl aldehyde presented herbal, fatty, and soap odors; and β-ionone showed floral and raspberry odors. Moreover, after fermentation, the OAVs of 1-octen-3-ol, hexanal, and trans-2,4-decadienal decreased to 0, and the OAV of 1-nonanal decreased from 43,093 to 10,541. This indicates that the fishy malodor of the fermented *S. japonica* seedling was dramatically reduced, while the floral and herbal notes were significantly enhanced.

**Figure 3:**
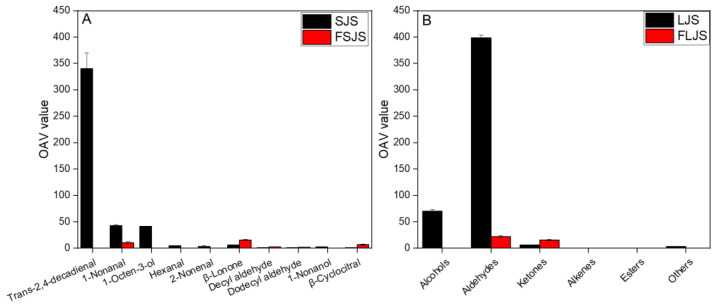
OAVs of the volatile compounds of the *S. japonica* seedling before and after *S. cerevisiae* fermentation. SJS means unfermented *S. japonica* seedling; FSJS means fermented *S. japonica* seedling (A refers to the OAV value of volatile compounds relate with sensory properties; B refers to the OAV value of alcohols, aldehydes, ketones, alkenes and esters).

Combining Figure 1 with Figure 3A, the OAV results are consistent with sensory properties. Unfermented *S. japonica* seedlings mainly present a fishy odor, which is reflected in OAV of trans-2,4-decadienal, 1-nonanal, and 1-octen-3-ol showing higher values. After fermentation, the sensory properties of the *S. japonica* seedling exhibited stronger notes of green, floral, sweet, and fruity. The significantly higher levels of β-ionone, β-cyclocitral, and decyl aldehyde in the OAV support their role as contributors to green, floral, sweet, and fruity notes.

Comparing the OAV before and after fermentation, as shown in Figure 3B, it is evident that alcohols, aldehydes, and ketones make significant contributions to the overall aroma. Aldehydes exhibit the highest OAV, indicating that aldehydes are the main contributors to the odor of the *S. japonica* seedling. After fermentation, the OAV of alcohols decreased to 0, while aldehydes decreased and ketones increased slightly. The change in the OAV of the *S. japonica* seedling after fermentation indicates that aldehydes and ketones are the primary contributors to the fermented *S. japonica* seedling odor. The OAV of alkanes, alkenes, and esters equals to 0, essentially indicating that these three compounds do not significantly contribute to the odor of the *S. japonica* seedling.

## 4. Discussion

It is important to eliminate the fishy odor in seafood, as this is a practical approach to enhance the flavor quality of seafood and encourage consumption [21,28,29,30]. The *S. japonica* seedling, which is the *S. japonica* harvested at the early growth stage and considered a new food material, was first investigated in terms of its aroma profile, aroma-active volatiles, and aroma enhancement through fermentation.

One of the objectives of this study was to identify the volatile compounds that contribute to the off-flavor of the *S. japonica* seedling. The result of this study, showing that the *S. japonica* seedling has 43 volatile compounds, is consistent with the findings of Zhu et al. [17]. Among these volatile compounds, 1-nonanal and hexanal were the most abundant in concentration. Furthermore, 1-nonanal, hexanal, and 1-octen-3-ol were the main contributors to the fishy odor of the *S. japonica* seedling. Nie et al. [16] reported that 1-octen-3-ol is a typical volatile compound contributing to the fishy odor in kelp. Peinado et al. [31] reported that hexanal and nonanal play an important role in determining the fishy odor of seaweed samples. Thus, this study identifies the presence of 1-octen-3-ol, trans-2,4-decadienal, 1-octen-3-one, and hexanal as responsible for the pronounced fishy odor in the *S. japonica* seedling, which is identical to the fishy odor reported in previous studies by Nie et al. [16] and Peinado et al. [31]. This is the first study on the volatile compounds of the *S. japonica* seedling that adds to our understanding of aroma volatile compounds in seafood.

The second aim of the study was to investigate the effectiveness of fermentation with *S. cerevisiae* in enhancing the aroma of the *S. japonica* seedling. Several groups of volatile compounds increased after fermentation, including β-ionone (108.4 μg/g), β-cyclocitral (20.5 μg/g), D-limonene (8.3 μg/g), dodecyl aldehyde (4.1 μg/g), and decyl aldehyde (7.1 μg/g). OAV results indicated that these volatile compounds contributed floral and fruity notes. The sensory characteristics of the fermented *S. japonica* seedling showed enhanced notes of green, floral, sweet, and fruity aromas. Thus, this study identifies β-ionone, β-cyclocitral, DL-limonene, dodecyl aldehyde, and decyl aldehyde as the key fragrance components in the *S. japonica* seedling. Liang et al. [32] reported that *Gracilaria lemaneiformis* produced floral and fruity aroma compounds after fermentation, with β-cyclocitral being the main aroma contributor. Xu et al. [23] reported that D-limonene is the main sweet contributor of *Bangia fusco-purpurea*. β-Ionone, β-cyclocitral, D-limonene, dodecyl aldehyde, and decyl aldehyde belong to terpenoids. The increase in terpenoids after fermentation may be due to the mevalonic acid (MVA) pathway in *S. cerevisiae*. The MVA pathway involves a series of enzymatic reactions starting with acetyl-coenzyme A (acetyl-CoA) and culminating in the production of isopentenyl diphosphate (IPP) and dimethylallyl diphosphate (DMAPP) [33,34]. Enzymes pivotal to this pathway include mevalonate kinase, phosphomevalonate kinase, and mevalonic acid dehydrogenase [35]. IPP and DMAPP are the basis for the synthesis of all terpenoid compounds [36].

The results showed that fishy odor contributors such as 1-octen-3-ol, trans-2,4-decadienal, 1-octen-3-one, and hexanal completely disappeared after fermentation with *S. cerevisiae*. Additionally, the concentration of 1-nonanal decreased from 47.4 ± 1.7 to 11.6 ± 1.5 µg/g. The results indicated that the fishy odor of the *S. japonica* seedling was dramatically weakened after fermentation with *S. cerevisiae*. This finding is consistent with the studies of Xu et al. [23] and Liang et al. [32], which demonstrated that fermentation with *Saccharomyces cerevisiae* effectively eliminated the fishy odor from *Bangia fusco-purpurea* and *Gracilaria lemaneiformis*, respectively, confirming the potential of *S. cerevisiae* fermentation to enhance the flavor profile of various seaweed species such as *S. japonica* seedlings. This finding is partially consistent with that of Zhu et al. [17], who found that the fishy odorant 1-octen-3-one disappeared from kelp after fermentation with *S. cerevisiae*, accompanied by the formation of 3-octanone. 1-Octen-3-ol, hexanal, heptanal, nonanal, and 2,4-heptadienal, which have been reported as the fishy odorants [16,23,31], are mainly derived from the oxidation of unsaturated fatty acids [37]. Unsaturated aldehydes and ketones, such as 1-octen-3-one, 2,6-nonadienal, 2,4-decadienal, and 2-nonenal, have been identified as the contributors to the fishy odor of kelp [17]. Microbial fermentation has been utilized to deodorize seaweed, fish, and other seafood [38,39,40], as enone reductase catalyzes the reduction of unsaturated bonds [17]. In addition, the reduction of fishy odor is related to the reactions of ester synthesis, dehydrogenation, reduction, and deformylation–oxygenation catalyzed by enone reductase, oxidases, aldehyde deformylating oxygenase, aldehyde dehydrogenase, ester synthase, aldoketo reductase, alcohol dehydrogenases, epoxide hydrolase, dehydrogenase, and acyltransferase [23]. Moreover, the disappearance of 1-octen-3-ol may be related to the catalysis of enzymes such as ADH, ALDH, ES, and ATF, which can convert 1-octen-3-ol to esters [41]. Enzymes could catalyze the hexanal to alcohol and esters through the reactions of reduction, dehydrogenation, and ester synthesis [42]. In addition, 1-nonanal could be converted to fatty acids and enol through ester synthesis, dehydrogenation, and reduction reactions in yeast [43]. Thus, the removal of the fishy odor and the enhancement of the overall aroma profile could be attributed to the bioreactions involving reduction, dehydrogenation, and ester synthesis during fermentation with *S. cerevisiae*. This will facilitate the understanding of how microorganism metabolism enhances the flavor of seaweeds, such as *S. japonica* seedlings. However, it is still unclear as to what the transformation pathways are for the eliminating or removing the fishy odor. Future research is required to explore how the fishy odors evolve during the fermentation process with *S. cerevisiae.*

## 5. Conclusions

The new food material *S. japonica* seedling was sensorily evaluated and found to have a strong fishy odor. Fermentation with *S. cerevisiae* is effective in eliminating the fishy note and enhancing the fruity, sweet, green, and floral notes of *S. japonica*. After fermentation, the number of volatile compounds decreased from 43 to 26; compounds such as 1-octen-3-ol, 2-octen-1-ol, hexanal, 2-nonenal, and 1-octen-3-one disappeared, while 1-nonanal decreased from 47.4 to 11.6 μg/g. On the contrary, some volatile compounds representing aroma increased after fermentation: β-ionone increased from 42.8 to 108.4 μg/g, β-cyclocitral from 3.8 to 20.5 μg/g, D-limonene from 0 to 8.3 μg/g, dodecyl aldehyde from 2.3 to 4.1 μg/g, and decyl aldehyde from 3.0 to 7.1 μg/g. The OAV revealed 14 major odor compounds (OAV ≥ 1), including 1-octen-3-ol, 1-nonanal, and trans-2,4-decadienal, which contribute to the strong fishy malodor; and β-ionone, β-cyclocitral, dodecyl aldehyde, and decyl aldehyde, which contribute to the floral and fruity aroma. After fermentation, the OAV of these fishy odorants decreases to 0, while the floral and fruity aroma increases. Fermentation with *S. cerevisiae* is a practical method to enhance the flavor quality of the new food material *S. japonica* seedling.

## Figures and Tables

**Table 1:** Quantitative analysis and approximate estimates of volatile compounds (relative to cyclohexanone) in the *S. japonica* seedling before and after *S. cerevisiae* fermentation.

NO.	RT/min	Compounds	RI ^a^	RI ^b^	Ion Fragment	Identification	Standard Curves	*R* ^2^	Concentration (µg/g)	
									SJS	FSJS
		Alcohols								
1	9.79	1-Octen-3-ol	979	976	57 72	MS, RI, Std	*Y* = 1.490988*X −* 0.071092	0.999	62.4 ± 3.0	-
2	11.37	2-Octen-1-ol	1067	1069	57 68	MS, RI, Std	*Y* = 6.551167*X −* 1.078406	0.999	4.6 ± 1.3	-
3	12.22	1-Octanol	1198	1194	79 91	MS, RI, Std	*Y* = 1.087064*X −* 7.655122	0.989	2.6 ± 0.4	-
4	16.18	Cis-Anethol	1214	1213	117 147 148	MS, RI, Std	*Y* = 1.325534*X* − 2.097888	0.998	0.5 ± 0.3	-
5	16.31	Trans-2-decen-1-ol	1265	1273	57 67	MS, RI, Std	*Y* = 3.258641*X* − 0.458222	0.994	32.1 ± 3.4	-
6	19.03	Phenethyl alcohol	1299	1277	57 71	MS, RI, Std	*Y* = 1.505271*X* − 1.243503	0.999	-	3.8 ± 1.1
7	25.08	1-Nonanol	1568	1569	55 56 70	MS, RI, Std	*Y* = 2.986852*X* − 1.702484	0.993	0.6 ± 0.1	-
8	27.50	Cedrol	1601	1601	95 150 222	MS, RI, Std	*Y* = 2.*743444X −* 1.758543	0.994	-	8.2 ± 0.2
		Aldehydes								
1	5.81	Hexanal	801	800	56 72	MS, RI, Std	*Y* = 15.83681*X* − 0.051104	0.995	24.5 ± 0.5	-
2	10.55	1-Nonanal	1103	1102	57 71	MS, RI, Std	*Y* = 6.034336*X* − 0.225634	0.993	47.4 ± 1.7 ^c^	11.6 ± 1.5 ^d^
3	10.68	2-Nonenal	1157	1158	55 70	MS, RI, Std	*Y* = 11.75698*X* − 0.063402	0.995	1.3 ± 0.7	-
4	10.93	2,4-Dimethylbenzaldehyde	1168	1175	105 133 134	MS, RI, Std	*Y* = 2.029053*X* − 3.532768	0.994	0.8 ± 0.1	-
5	13.50	Oct-2-Enal	1192	1196	41 81 110	MS, RI, Std	*Y* =1.023356*X* − 5.775350	0.999	2.8 ± 0.6 ^c^	12.0 ± 2.2 ^d^
6	14.93	Decyl aldehyde	1204	1204	57 70	MS, RI, Std	*Y* = 1.649864*X* − 3.354954	0.997	3.0 ± 0.8 ^c^	7.1 ± 0.4 ^d^
7	15.59	β-Cyclocitral	1212	1214	109 137 152	MS, RI, Std	*Y* = 3.236918*X* + 0.228739	0.993	3.8 ± 0.3 ^c^	20.5 ± 2.9 ^d^
8	18.94	2,6,6-Trimethyl-1-Cyclohexene-1-Acetaldehyde	1248	1251	107 151 166	MS, RI	Estimated	-	1.3 ± 0.4 ^c^	8.1 ± 1.2 ^d^
9	19.25	Undecanal	1305	1308	57 82	MS, RI, Std	*Y* =1.493987*X* − 0.218564	0.996	0.9 ± 0.1	-
10	25.06	Trans-2,4-decadienal	1403	1402	81 95 152	MS, RI, Std	*Y* = 2.045690*X* + 0.061021	0.999	3.4 ± 0.3	-
11	26.03	Dodecyl aldehyde	1412	1412	57 82	MS, RI, Std	*Y* = 0.743598*X* − 0.101732	0.993	2.3 ± 0.6 ^c^	4.1 ± 0.4 ^c^
		Ketones								
1	10.13	1-Octen-3-one	973	974	30 41 43	MS, RI, Std	*Y* = 0.396353*X* − 3.222123	0.999	7.1 ± 0.1	-
2	10.15	2,2,6-Trimethylcyclohexanone	1030	1027	56 82 140	MS, RI, Std	*Y* = 3.682041*X* − 1.250163	0.993	49.3 ± 1.7	-
3	11.59	3-Methyl-2-cyclohexen-1-one	1053	1055	54 82 110	MS, RI, Std	*Y* = 0.721058*X* − 3.702106	0.999	19.3 ± 0.4	-
4	11.89	3,5-Octadien-2-one	1090	1081	81 95 124	MS, RI, Std	*Y* = 4.146752*X* − 0.034237	0.995	8.1 ± 2.2	-
5	12.15	3-Acetyl-2-octanone	1114	1118	54 82 138	MS, RI, Std	*Y* = 2.084362*X* − 0.239612	0.994	1.3 ± 0.5	-
6	12.74	2,6,6-Trimethyl-2- cyclohexanedione	1139	1139	68 96 152	MS, RI	Estimated	-	3.8 ± 0.4	-
7	21.85	2-Methyloctan-3-one	1400	1407	58 71 198	MS, RI, Std	*Y* = 6.201378*X* − 0.920482	0.998	3.0 ± 0.3 ^d^	1.3 ± 0.6 ^d^
8	21.92	β-Ionone	1415	1412	135 177 192	MS, RI, Std	*Y* = 4.936785*X* − 0.014371	0.997	42.8 ± 3.1 ^c^	108.4 ± 5.6 ^d^
9	22.14	4-(2,6,6-trimethylcyclohexen-1-yl) butan-2-one	1425	1433	121 161 194	MS, RI	Estimated	-	2.8 ± 0.7	-
10	23.23	5,9-Undecadien-2-one, 6,10-dimethyl-	1443	1451	69 136 194	MS, RI	Estimated	-	6.3 ± 0.8	-
		Alkanes						-		
1	21.64	n-Pentadecane	1499	1500	57 71 212	MS, RI	Estimated	-	2.0 ± 0.2 ^c^	5.2 ± 1.7 ^c^
2	21.82	n-Hexadecane	1600	1600	57 71 226	MS, RI	Estimated	-	0.9 ± 0.1 ^c^	4.7 ± 1.5 ^d^
3	21.87	n-Heptadecane	1700	1700	57 71 240	MS, RI	Estimated	-	37.8 ± 8.5 ^c^	23.3 ± 4.2 ^d^
4	24.97	n-Dodecane	1199	1200	57 71 170	MS, RI	Estimated	-	-	6.1 ± 2.0
5	26.55	Iodoheptane	1261	1260	57 71 85	MS, RI	Estimated	-	5.2 ± 1.0	-
6	27.13	2,6,11-Trimethyldodecane	1274	1275	57 71 212	MS, RI	Estimated	-	-	20.7 ± 4.2
7	27.94	Butyl-nonane	1282	-	57 71 184	MS, RI	Estimated	-	-	2.5 ± 1.4
8	28.68	4,6-Dimethyl dodecane	1320	1325	57 71 113	MS, RI	Estimated	-	-	7.6 ± 2.3
9	32.01	Tetradecane	1399	1400	57 71 198	MS, RI	Estimated	-	-	3.8 ± 1.4
		Alkenes								
1	4.05	5,5-Dimethyl-2-ethyl-1,3-cyclopentadiene	835	840	79 107 122	MS, RI	Estimated	-	5.4 ± 1.3	-
2	8.32	3,5,5-Trimethyl-1-hexene	967	968	57 70 126	MS, RI, Std	*Y* = 0.985246*X* − 0.512014	0.999	3.9 ± 0.6	-
3	8.91	3,5,5-Trimethyl-2-hexene	971	985	57 72	MS, RI, Std	*Y* = 2.150213*X* − 3.251024	0.997	68.4 ± 10.4	-
4	10.13	Limonene	997	995	68 93 136	MS, RI, Std	*Y* = 1.494374*X* − 0.117216	0.998	7.4 ± 1.0	-
5	10.65	α-Terpinene	1012	1014	55 69	MS, RI, Std	*Y* = 2.*10673X −* 0.0584598	0.998	10.4 ± 0.4	-
6	29.87	3-Methyl-6-(1-methyl vinyl)-cyclohexene	1680	1680	55 69 238	MS, RI	Estimated	-	13.7 ± 20.7	-
7	30.28	1,3-Octadiene	1692	1692	55 97 238	MS, RI, Std	*Y* = 6.231025*X* − 0.045231	0.999	7.8 ± 0.2	-
8	34.69	D-limonene	1729	1728	68 93 136	MS, RI, Std	*Y* = 6.131623*X* − 3.776805	0.997	-	8.3 ± 3.5
9	37.83	1-Undecene	1791	1791	55 69 97	MS, RI, Std	Estimated	-	-	3.2 ± 0.3
10	38.23	4-Methyl-1-hendecene	1799	1785	57 71	MS, RI, Std	*Y* = 0.453254*X* − 4.741452	0.995	-	15.5 ± 2.6
11	40.28	Heptadecene	1821	1822	55 97 238	MS, RI, Std	*Y* = 4.965865*X* − 0.552135	0.998	31.0 ± 3.1	-
		Esters								
1	12.52	Hexyl acetate	1004	1004	43 56 84	MS, RI, Std	*Y* = 0.886523*X −* 2.443578	0.999	5.1 ± 1.3	-
2	16.87	Phenethyl acetate	1283	1281	43 91 105	MS, RI, Std	*Y* = 3.412776*X* − 0.743251	0.999	-	8.7 ± 2.4
3	18.12	Hexyl butyrate	1282	1284	33 61 92	MS, RI, Std	*Y* = 5.120312*X* − 0.635227	0.996	0.6 ± 1.5	-
4	18.43	Ethyl caprate	1289	1287	43 58 97	MS, RI, Std	*Y* = 1.523712*X* − 0.641224	0.998	-	2.3 ± 1.2
5	23.31	Ethyl laurate	1724	1725	45 63 112	MS, RI, Std	*Y* = 3.265843*X* − 0.536241	0.999	-	3.6 ± 1.4
6	32.03	Methyl palmitate	1827	1827	43 60 102	MS, RI, Std	*Y* = 4.776543*X* − 3.156005	0.993	65.3 ± 8.2 ^c^	44.3 ± 10.7 ^d^
		Others								
1	9.62	2-Pentylfuran	988	987	81 138	MS, RI, Std	*Y* = 1.063317X − 0.033126	0.994	18.9 ± 3.1	-
2	18.81	2-Methyl-2-allylphenol	1343	1349	105 133 148	MS, RI	Estimated	-	-	2.7 ± 0.6
3	22.16	1,6-Dimethylnaphthalene	1503	1502	141 155 156	MS, RI	Estimated	-	0.4 ± 0.5	-

Note: SJS refers to unfermented *S. japonica* seedling; FSJS refers to fermented *S. japonica* seedling; RT represents the retention time of aroma compounds on the Rtx-5MS column. RI ^a^ was calculated based on the retention time of C_8_–C_20_. RI ^b^ was obtained from the web: http://webbook.nist.gov/chemistry/ (accessed on 20 March 2020). Identification method: MS, mass spectrum comparison using (NIST11, NIST11s, FFNSC1.3) library; RI: retention index in agreement with literature value; Std: validated with authentic standards. Estimations suggest that the unquantified volatile compounds were treated as equivalent units of internal standard added to a sample matrix. ^c,d^ Signal intensities are shown as mean ± standard deviation (*n* = 3). Significant differences were performed using Tukey’s test (*p* < 0.05) in SPSS 19.0.

**Table 2:** OAVs of the volatile compounds of the *S. japonica* seedling before and after fermentation.

No.	Volatile Compounds	Odor Description ^a^	Threshold Value ^b^ (μg/kg)	OAV Value	
				SJS	FSJS
1	1-Octen-3-ol	Mushroom, fatty, fishy	1.5	41,603 ± 1.995	-
2	2-Octen-1-ol	Mushroom, fatty	840	5 ± 1.538	-
3	1-Octanol	Lemon, floral	0.13	20 ± 3.204	-
4	1-Nonanol	Earthy, fatty	280	2 ± 0.355	-
5	Hexanal	Herbal, fishy	5	4895 ± 0.104	-
6	1-Nonanal	Herbal, paint, fatty, fishy	1.1	43,093 ± 1.628 ^c^	10,541 ± 1.355 ^d^
7	2-Nonenal	Fishy, herbal	0.4	3253 ± 1.751	-
8	2,4-Dimethylbenzaldehyde	Bitter Almond	200	4 ± 0.500	-
9	Decyl aldehyde	Ocean, cucumber, herbal	1.97	1.11 ± 0.30 ^c^	2.615 ± 0.138 ^d^
10	β-Cyclocitral	Liquorice, fruity, fresh	3	1274 ± 0.103 ^c^	6831 ± 0.974 ^d^
11	Undecanal	Fruity	12.5	72 ± 0.004	-
12	Trans-2,4-decadienal	Fishy, fatty	10	340 ± 30.002	-
13	Dodecyl aldehyde	Herbal, fatty, soap	2	1150 ± 0.295 ^c^	2050 ± 0.201 ^d^
14	3,5-Octadien-2-one	Woody, sweet	150	54 ± 0.006	-
15	β-Ionone	Floral, raspberry	2	21,431 ± 0.441 ^c^	54,243 ± 0.803 ^d^
16	D-Limonene	Lemon, citrusy	200	-	42 ± 0.015
17	Methyl palmitate	Fatty	2000	33 ± 0.006 ^c^	22 ± 0.013 ^d^
18	2-Pentylfuran	Beany, spicy	5.8	3204 ± 0.521	-

Note: SJS refers to unfermented *S. japonica* seedling; FSJS refers to fermented *S. japonica* seedling. ^a^ Odor description refers to the Odor Database (http://www.odour.org.uk/). ^b^ Threshold values were according to the reported compilations of odor threshold values in air, water, and other media [25] ^c,d^ Signal intensities were shown in mean ± standard deviation (*n* = 3). Significant differences were performed using Tukey’s test (*p* < 0.05) in SPSS 19.0.

## Data Availability

The original contributions presented in the study are included in the article, further inquiries can be directed to the corresponding author.
